# Disclosure of abuse among female patients within general psychiatric care - a cross sectional study

**DOI:** 10.1186/s12888-016-0789-6

**Published:** 2016-03-24

**Authors:** K. Örmon, C. Sunnqvist, C. Bahtsevani, M. Torstensson Levander

**Affiliations:** Department of Care Science, Faculty of Health and Society, Malmö University, 205 06 Malmö, Sweden; Department of Criminology, Faculty of Health and Society, Malmö University, Malmö, Sweden

**Keywords:** Adult survivors of abuse, Domestic violence, Intimate partner violence

## Abstract

**Background:**

Experiences of abuse are common among women in general psychiatric care. Even so, there are to our knowledge no previous national or international studies exploring disclosure in a general psychiatric setting of female patient’s experiences of abuse to staff or to formal and informal networks.

This study aimed to explore women’s disclosure of experiencing physical, emotional and/or sexual abuse during their most recent contact with staff at a general psychiatric clinic. The study also aimed to determine whether the women have previously disclosed abuse to anyone.

**Methods:**

A consecutive sampling of eligible female patients at a general psychiatric clinic in an urban area of southern Sweden answered the NorVold Abuse Questionnaire, NorAQ, a self-administrated questionnaire. NorAQ has previously been used and further developed to compare the prevalence of abuse in women present in gynecological outpatient settings in the five Nordic countries. Seventy-seven women with experiences of abuse participated in the research. Descriptive statistics were used to analyse the data.

**Results:**

Most respondents did not disclose their experiences of abuse to staff at the general psychiatric clinic. Women with experiences of physical abuse (*n* = 40), emotional abuse (*n* = 37) and sexual abuse (*n* = 37) chose not to disclose their experiences. Respondents disclosed abuse more often to others than to staff.

**Conclusions:**

Our findings indicated the importance of including routine questions concerning abuse experiences as a natural part of female patients’ medical history.

## Background

According to the first national study concerning violence against women in Sweden, 46 % of all women in Sweden have experienced physical or/and sexual violence or/and verbal threats from a man after the age of 15 [1]. Another Swedish population-based study reported that 46 % of women have experienced severe sexual, physical or emotional violence sometime during their life course [2].

One consequence of intimate partner violence was the association between experiences of abuse and women’s mental ill health. Research has shown a high prevalence for depression, post-traumatic stress disorder (PTSD), anxiety, suicide and deliberate self-harm [3]. A review exploring gender differences and how mental healthcare services should respond to domestic violence, reports an association between drug use and alcohol abuse, suicidal behaviour, sleeping disorders, eating disorders and domestic violence [4]. Being abused as a child also has consequences for mental ill health as an adult and is associated with depression, anxiety and substance abuse [5]. Extensive research has also associated childhood abuse with cluster b personality disorders, attachment disorders and PTSD. Moreover, personality dysfunction is one of the most common presentations to psychiatric settings. A review based on 44 studies reported association between early life stress, such as experiences of abuse and neglect and mood disorder, anxiety disorders and personality disorders during adulthood. The sexual abuse was associated with personal disorder especially cluster B [6].

Research has shown that a large number of female patients in psychiatric care have experienced domestic violence [7]. Almost one out of three women within psychiatric settings has experienced domestic violence. A review by Oram et al. (2013) based on 42 articles reported a high lifetime prevalence of domestic violence among women within psychiatric in-patient care (30 %) and for psychiatric out-patient care the prevalence was slightly higher (33 %). A Swedish study found a high prevalence of abuse in female users of psychiatric services, reporting a 53 % prevalence of childhood abuse and a 63 % prevalence of abuse during adulthood [8]. Another study reported that 20 % of general psychiatric patients had been victimized within 1 year prior to participation in the study; the research found it is more common for victimized women to seek psychiatric care than for women with no experience of violence [9]. A study conducted in psychiatric out-patient care reported that 67 % of the male and female patients with psychosis had experienced violence during their childhood. One third of the patients reported experiences of abuse during previous year [10]. A Swedish study showed that more than half of the women in a psychiatric setting had endured abuse during their childhood. The most common abuse was emotional abuse (33 %) followed by sexual abuse (28 %) and physical (24 %). More than half of those women (53 %) had been exposed to more than one form of abuse and almost three of four girls had been abused repeatedly during their childhood (71 %). A parent or sibling was the most frequent perpetrator (63 %) [11].

Health-care providers must routinely ask questions regarding experiences of partner violence. Intimate partner violence was often under-detected in psychiatric settings, which could be exacerbated by the women not receiving adequate care and support [12]. If staff at a general psychiatric clinic are not aware of a patient’s history of abuse, the risk of not receiving adequate treatment and support increases and thereby affects the patient’s wellbeing.

There are a variety of psychosocial factors that determine if patients’ experiences of abuse are identified and clinically addressed by health care workers. Nurses in a Finnish government health organisation pointed out it is a necessity for abused women to meet nurses regularly before being able to talk about experienced intimate partner abuse [13]. Another study reported how hospital-based nurses were more reluctant than community-based nurses to address abuse unless women brought up the issue themselves [14]. A Swedish study highlighted that staff in welfare services working with people with mental illness care hold inadequate knowledge to be able to identify and interact with abused women with mental illness. They possessed inadequate knowledge of abuse, and abuse related sequelae, to be able to identify and appropriately work with women who have abuse histories [15].

There are a number of reasons why abused women choose to disclose or not disclose their experiences of abuse. One study showed that health care personnel seldom ask questions regarding domestic violence [16]. Factors that could prevent disclosure were self-blame and blaming attitudes from others, fear of violent acts, social services and for not being believed [17]. Judgmental attitudes from staff or the presence of a perpetrator at the hospital or clinic could also prevented women from disclose experiences of abuse. Healthcare staff ignoring the abuse as well as emotional distress was other reasons for not disclosing abuse [18]. Environmental factors such as security, facilities and information about domestic violence are other factors that could prevent disclosure [19]. Factors such as lack of time to raise the issue, no privacy and no continuity of care could also prevent abused women from disclosing domestic violence to health professionals [20]. A review regarding experiences of healthcare and adult survivors of child sexual abuse, reported a need to create an open and sensitive atmosphere with no judgmental or victim blaming. It was also evident that there were sufficient time for disclosing the abuse, and that the person was believed when talking about experiences of abuse [21].

The present study aims to explore women’s disclosure of experiencing physical, emotional and/or sexual abuse to staff during their most recent contact with staff at a general psychiatric clinic. In addition, the study aims to determine if the women have ever disclosed experiences of abuse to anyone.

## Methods

A cross-sectional design was used to gather data from women in a general psychiatric setting regarding experiences of abuse. A consecutive sampling method was applied to eligible women visiting a general psychiatric clinic was conducted in from 1 September to 31 October 2010. The questions describing disclosure of abuse were part of the NorVold Abuse Questionnaire (NorAQ), which has previously been used and further developed to compare the prevalence of abuse in women present in gynaecological outpatient settings in the five Nordic countries [22].

The present study involved women at general psychiatric clinics, with three outpatient and four inpatient general psychiatric units in an urban area of southern Sweden. Most women at the clinic are treated for mental health issues such as affective disorders, suicidal behaviour, anxiety disorders, personality disorders and eating disorders. The most common languages spoken among the female patients at the clinic were Swedish, Arabic and Serbo-Croatian; other languages were thereby excluded from the study. During the period of data collection 450 female patients agreed to participate in the study. Of the 450 participants, 88 returned the questionnaire and 77 women had self-reported abuse experiences at some point during the course of their lives, these women were therefore included in the study. Of the seventy-seven women who participated in the study, 58 % (*n* = 45) were visiting as an outpatients and 42 % (*n* = 32) were inpatients. Fifty-three percent (*n* = 41) had experienced each of the three forms of abuse (i.e., physical, emotional and sexual) at some point during their life course. Only 6 % (*n* = 5) of the women had endured one kind of abuse during childhood, adulthood and/or during their lifetime. Of the seventy-seven participants, fifty-two women self-reported affective disorders, suicidal behaviour and/or anxiety disorders as reasons for seeking general psychiatric care at that particular time; five women self-reported eating disorders; two suffered from AD/HD; three women self-reported paranoid and/or psychotic behaviour; five women self-reported contact due to needing support, therapy or to renew a prescription; seven women stated abuse as a reason for seeking care; and three women did not reply to the question (see Table 1 for demographic facts).

**Table 1:** Demographics regarding participating women (*n* = 77)

Mean age	34 ± 11 years
Country of birth	
Sweden	65 (84 %)
Other	12 (16 %)
Years of education	
9 years or less	11 (14 %)
10–12 years	17 (22 %)
13 years or more	49 (64 %)
Marital status	
Single	33 (43 %)
Regular partner	38 (49 %)
Other	6 (8 %)
Income source	31 (40 %)
Employed	7 (9 %)
Unemployed or employment training courses	
Student	9 (12 %)
Sick leave	19 (25 %)
Other	8 (10 %)
Unknown	3 (4 %)
Experience of abuse	
Emotional	61 (79 %)
Physical	72 (93 %)
Sexual	55 (71 %)

Due to the sensitive subject matter, it was important the participants could answer the questionnaires themselves. Any assistance from the staff could influence the accuracy of the women’s answers. Participants from the inpatient units were excluded if they had symptoms of confusion (*n* = 7), an intellectual disability (*n* = 2) or visually disabled (*n* = 2). Women who were transferred to other units before receiving the questionnaire (*n* = 9) were also excluded. Exact details of those excluded from the three outpatient units were not collected due to the questionnaire distributed at the reception desk or by non-clinical office staff. The completed questionnaires were returned by mail in prepaid envelopes or in mailboxes at the psychiatric units. The first author visited the inpatient units and one of the outpatient units during weekday mornings and distributed the questionnaires in person to the female patients. At two of the outpatient units, respondents received the questionnaire by staff at the reception desk; the women were verbally informed about the purpose of the study upon receiving the questionnaires. At each inpatient unit, a nurse evaluated the women’s mental status on a daily basis, before asking them if they were prepared to participate in the study. Women with a high level of anxiety or suicidal ideation were asked when stabilized.

In the present study, the NorVold Abuse Questionnaire, NorAQ, was used. NorAQ is a self-administrated questionnaire wherein the experiences of various forms of abuse are dichotomous by answering yes or no to questions on experiencing physical, emotional or sexual abuse [15]. The abuse-related questions in NorAQ have been tested against the Conflict Tactic Scale, the Sexual Abuse Questionnaire and face-to-face interviews. NorAQ performed better against abuse related interviews than against the Sexual Abuse Questionnaire, and it equaled the performance of the Conflict Tactic Scale. Test-retest reliability ranged from 84 % to 95 %. Responses to the questions concerning emotional and physical abuse were ‘mild’, ‘moderate’ and ‘severe’ [23]. The question in NorAQ, regarding disclosure of experiencing abuse at the most recent contact with staff at the general psychiatric clinic was as follows: ‘Recall your most recent contact with staff at the general psychiatric clinic: Did you tell anyone at the clinic about you being subjected to physical/emotional/sexual abuse?’ The response alternatives were ‘No’; ‘Yes, he/she knew already’; ‘Yes, when he/she asked about it’; and ‘Yes, I told him/her spontaneously’. The other questions covered disclosure of experiences of abuse to someone, and the response alternatives were as follows: ‘No’; ‘Yes, partly’; and ‘Yes, about all of it’. K. Swahnberg, coordinator of the NorVold Abuse Questionnaire research network, granted permission to use and modify NorAQ. Questions regarding disclosure of abuse were modified using the term ‘general psychiatric clinic’. In this paper, abuse is presented as physical, emotional or sexual. Registered Nurses (RN: s), nurses specialized in psychiatric care, psychiatrists, nurse aids and counsellors are referred to as ‘staff’.

### Data analysis

Descriptive statistics were used to describe the disclosure of physical, emotional or sexual abuse either at their most recent contact with staff at the general psychiatric clinic or when previously having talked to someone about experiencing abuse. The questions concerning physical, emotional and sexual abuse were dichotomised. The Statistical Package for the Social Sciences, Version 20 (SPSS, Chicago, IL), was used to analyze the statistical data.

### Ethics

The participants were given verbal and written information that their participation was voluntary and that they had the right at any time and without cause or explanation to decline or cease participation. All participants were guaranteed confidentiality, and declining to participate would not affect the care provided to the women. A written consent was obtained. Together with the NorAQ questionnaire, they received the phone number to *Kvinnofridslinjen,* a national helpline for women subjected to threats or violence. The Regional Ethical Review Board in Lund (Dnr: 2010/3) approved the study. Staff at the general psychiatric clinic was informed of the risk of discomfort after participation, and the first author (K.Ö) visited the inpatient units in the mornings from Monday to Friday and was thereby able to answer any questions staff or patients may have had. These precautions aimed to make the women feel safe and motivated to answer questions concerning disclosure of abuse. Staff at the units involved in the study had also been informed verbally and in writing about the study. They were thus aware that the respondents might experience discomfort and a need of support. An envelope containing the research material was given to each participant, and no reminders were sent to the women’s home addresses due to the possible risk of abuse. The participants opened the envelope and answered and the questionnaire privately, returning their response at a time of their choosing.

## Results

The results show that according to the questions asked in the questionnaire, a majority of the women in the study chose not to disclose experiences of abuse to staff at the general psychiatric clinic. Experiences of physical abuse was the most common form of abuse experienced by respondents (93 % *n* = 72), but more than half of the women did not talk to the general psychiatric clinic staff about their experiences (*n* = 40). A majority of the women talked to others then staff at the general psychiatric clinic about their experiences of physical abuse (*n* = 62) (Table 2).

**Table 2:** Disclosure of abuse among female patients in general psychiatric care (n=77)

Disclosure of abuse during most recent contact with staff at a general psychiatric clinic (*n* = 77)	Disclosure of Physical abuse (*n* = 72)	Disclosure of emotional abuse (*n* = 61)	Disclosure of sexual abuse (*n* = 55)
No	40 (55 %)	23 (38 %)	37 (67 %)
Yes, he/she knew already	2 (3 %)	2 (3 %)	3 (5 %)
Yes, when he/she asked about it	9 (12 %)	17 (28 %)	6 (11 %)
Yes, I Told him/her spontaneously	18 (25 %)	18 (29 %)	7 (13 %)
Unknown	3 (4 %)	1 (2 %)	2 (4 %)
Disclosure of abuse to others then staff at the most recent contact in a general psychiatric clinic (*n* = 77)			
No	8 (11 %)	3 (5 %)	11 (20 %)
Yes, partly	44 (61 %)	44 (72 %)	34 (62 %)
Yes, about all of it	18 (25 %)	14 (23 %)	10 (18 %)
Unknown	2 (3 %)	0 (0 %)	0 (0 %)

Seventy-nine percent of the women (*n* = 61) had experienced emotional abuse. They disclosed their experiences to general psychiatric clinic staff more frequently (*n* = 37), but it was more common for them to talk to others than to staff working at the general psychiatric clinic (*n* = 58). Sexual abuse was the least reported type of abuse. It was experienced by 71 % (*n* = 55) of the women; most respondents had not discussed their sexual abuse with anyone on staff during their most recent contact at the general psychiatric clinic (*n* = 37). In comparison, 44 women had revealed the experience of sexual abuse to others. Most of the staff at the general psychiatric clinic was not aware according to the participants’ data, that the women had experienced physical, emotional and/or sexual abuse sometime during the course of their lives (Table 2).

## Discussion

The study’s results showed that women who had experienced abuse often choose not to disclose their experiences of abuse to staff at a general psychiatric clinic. One important aspect is that abused women may not always identify themselves as abused due to the process of normalisation of the experienced violence. Another noteworthy point is that healthcare sector staff presumes that abused women do not hesitate to disclose that they are living in an abusive relationship [24]. The result also showed that only a few members of staff were informed face-to-face by the women about their experiences of abuse. When staff in general psychiatric settings are not aware of female patients’ experiences of abuse, one risk is that symptoms of abuse could be mistaken for symptoms of mental ill health [25]. By not separating “symptoms of abuse” from symptoms of mental ill health, patients will not receive adequate care and support. Another factor that could prevent women from disclosure is the public debate in the Nordic countries that highlights the rational behavioral imperative to leave a relationship after ‘the first abusive incident’; this argument has a tendency to make women feel ‘stupid’ for not leaving abusive relationships [26].

The study’s results showed that women who had experienced abuse prefered to disclose their experiences to others than to staff at the general psychiatric clinic. One study demonstrated that abused women needed the informal support of friends and family prior to asking for professional support [27]. By sharing their problems with family and friends the women felt relieved as well as strengthened to seek formal support [27]. Similarly, other research has revealed that women prefer to disclose experiences of violence to family and friends prior to healthcare professionals [28, 29]. This is in line with our results, where most women who disclosed the abuse chose to disclose it to somebody other than the general psychiatric clinic staff.

By using the NorAQ questionnaire, explicit information regarding abused women’s disclosure of abuse to staff and others was obtained, and an important issue concerning psychiatric care was highlighted. To increase knowledge of experiences of abuse in a general psychiatric setting, women who participated in the present study were asked to further participate in a further qualitative study, which aimed to elucidate how women subjected to physical, emotional and/or sexual abuse experienced the care provided at a general psychiatric clinic after the disclosure of their abuse experiences. The study’s results based on interviews with 10 women, revealed that the participants experienced the care received as both caring and non-caring after their disclosure of abuse to the staff at the general psychiatric clinic. The non-caring care was experienced when there was a strong focus on the diagnosis of the psychiatric illness which served to burden the participants with guilt and misbeliefs after the disclosure of abuse. The experiences of when a caring environment was present came forward in relation to when the participants felt they were acknowledge by the staff and when the disclosing of the abuse also had a meaning and influenced the care given [30].

One important result of this research is that according to the participants, few staff members were aware of the women’s history of abuse, and, when disclosure occurred, it was more common for the women to speak out spontaneously rather than after being asked by staff. An important question emerges from this finding; were the abused women in our study more secure about raising the topic than the staff? A review based on eleven interview and/or self-administrated questionnaires showed that staff in acute mental health settings do not routinely ask questions regarding experiences of sexual abuse during childhood to adult patients [31]. The staff felt it intrusive and feared that asking about experiences of sexual abuse during childhood could worsen the patients’ mental health. Other reasons were lack of recourses or that the issue was irrelevant [31]. Another review based on 13,027 women in 11 trials conducted in healthcare settings that assessed the effectiveness of screening for intimate partner violence showed that screening increased detection of abuse among female patients within the healthcare sector. However, research also clarifies both that screening itself does not decrease violence over time and that there is a need for further interventions [32]. The result of the study highlights the need to implement policy and clinical procedural guidelines regarding identification of abuse in psychiatric care. It is also important that all clinicians working in general psychiatric settings are educated in asking questions concerning abuse.

### Limitations of the study

One limitation of the study is the response rate. Reasons for not participating could be that the women had no experiences of abuse, or, as one women said, ‘I don’t want to go back there. … That is history; I want to focus on my health’. Another reason could be that women with severe mental disorders or in critical states probably chose not to participate. Other reasons for not participating could be due to the experiences of abuse: It is not uncommon that women who have experienced abuse do not consider themselves as ‘abused women’ either because of the process of normalisation of the experienced violence or because their experiences are not processed [1].

Another limitation is a lack of information regarding the women’s relations with the staff. A sample of 77 women is small, and thus cannot be said to be representative for women in general psychiatric care. The descriptive nature of the study, and therefore, its inability to detect causal linkages is also considered a limitation. The results of this study should not be used to make generalizations; nevertheless, the study contributes to the body of knowledge regarding women’s disclosure of abuse. Further research is required on the reason why women who have experienced abuse choose not to disclose their experiences to staff working in general psychiatric care.

## Conclusions

Mental health issues are more common among women with experiences of abuse than among women without abuse experiences. Despite this reality, and the biopsychosocial risks associated with it, research confirms that many female patients within psychiatric care has experienced abuse sometimes during their life course. To be able to care for these women and provide them with appropriate care, support and treatment routine questions regarding experiences of abuse are required within psychiatric care delivery. The majority of the women in the study chose not to disclose their experiences of abuse to staff at the general psychiatric clinic; this indicates that a significant component of women’s clinical picture may be missing. The results also demonstrated how women preferred talking to others more than to staff about their abuse experiences. Our findings indicate the importance of including questions concerning abuse as a natural part of patients’ medical history. In line with the present findings, research concerning women’s experiences of abuse in general psychiatric care should be further explored and should be researched with a larger sample size together with a mixed methods research approach.

### Relevance to clinical practice

To be able to care for and treat patients in general psychiatric care, important questions regarding experiences of abuse need to be asked. Questions regarding experiences of abuse should be included within patients’ medical histories and guidelines to identify and care for abused women should be implemented in psychiatric care.

## Declerations

### Ethics approval and consent to participate

The Regional Ethical Review Board in Lund (Dnr: 2010/3) approved the study and the participants signed a form of consent before participation.

### Consent to publish

Consent to publish is not required.

### Availability of data and materials

By contact with corresponding author.
